# Multiorgan Imaging for Interorgan Crosstalk in Cardiometabolic Diseases

**DOI:** 10.1161/CIRCRESAHA.125.325517

**Published:** 2025-05-23

**Authors:** Ana Devesa, Victoria Delgado, Ladislav Valkovic, Joao A.C. Lima, Eike Nagel, Borja Ibanez, Betty Raman

**Affiliations:** Centro Nacional de Investigaciones Cardiovasculares, Madrid, Spain (A.D., B.I.).; Non-Invasive Cardiovascular Imaging Section, Heart Institute, University Hospital Germans Trias i Pujol, Badalona, Spain (V.D.).; Centre of Comparative Medicine and Bioimaging, Institute of Research Germans Trias i Pujol, Badalona, Spain (V.D.).; Division of Cardiovascular Medicine, Radcliffe Department of Medicine, Oxford Centre for Clinical Magnetic Resonance Research, University of Oxford, United Kingdom (L.V., B.R.).; Department of Imaging Methods, Institute of Measurement Science, Slovak Academy of Sciences, Bratislava (L.V.).; Department of Medicine and Radiology, Johns Hopkins Hospital, Baltimore, MD (J.A.C.L.).; Institute for Experimental and Translational Cardiovascular Imaging, Goethe University Frankfurt am Main, Germany (E.N.).; German Centre for Cardiovascular Research (DZHK), Partner Site Rhine-Main, Frankfurt, Germany (E.N.).; Cardiology Department, IIS-Fundación Jiménez Díaz University Hospital, Madrid, Spain (B.I.).; CIBERCV, Instituto de Salud Carlos III, Madrid, Spain (B.I.).

**Keywords:** acute coronary syndrome, atrial fibrillation, bone marrow, cardiovascular diseases, insulin resistance

## Abstract

Cardiometabolic diseases encompass a group of conditions characterized by metabolic and inflammatory abnormalities that increase the risk of diabetes and cardiovascular disease. These syndromes involve multiple organs, including the heart, arterial system, brain, skeletal muscle, adipose tissue, hematopoietic system, liver, kidneys, and pancreas. The crosstalk between these organs contributes to the development of disease. Advances in imaging techniques, such as magnetic resonance imaging, magnetic resonance spectroscopy, computed tomography, and positron emission tomography, have revolutionized the evaluation of these conditions. Hybrid imaging modalities, such as positron emission tomography/computed tomography and positron emission tomography/magnetic resonance imaging, provide unique insights into the anatomy and metabolic alterations occurring in response to cardiometabolic risk factors. These methods are particularly valuable for assessing multisystemic involvement and interorgan crosstalk, revealing critical interactions such as the brain-heart axis, the heart-liver axis, and the fat-muscle-heart dynamics. This review discusses the role of state-of-the-art imaging techniques in evaluating the pathophysiological mechanisms underlying these complex syndromes and the clinical applications of the different imaging techniques in the assessment of cardiometabolic diseases.

The concept of cardiometabolic disease encompasses a group of conditions involving cardiovascular, renal, metabolic, and inflammatory abnormalities.^1,2^ Metabolic syndrome (MetS), characterized by central obesity, hypertension, dyslipidemia, and insulin resistance, represents a key contributor to cardiometabolic diseases, significantly increasing the risk of type 2 diabetes and cardiovascular events.^3^

The spectrum of cardiovascular diseases associated with MetS is broad, ranging from vascular events such as acute coronary syndrome and cerebrovascular accidents to myocardial abnormalities and heart failure (HF).^4^ MetS and diabetes are linked to an elevated risk of atherosclerotic diseases, including coronary artery disease (CAD), cerebrovascular disease, and peripheral arterial disease.^5,6^ Furthermore, these conditions affect not only the macrovascular system but also the microvascular network, as evidenced by alterations in coronary,^7,8^ cerebral,^9^ and renal^10^ microcirculation, among other organs. Even in patients without diabetes, MetS and insulin resistance are associated with changes in myocardial metabolism, reducing cardiac efficiency; notably, cardiac changes can be observed much earlier than the appearance of any events.^11^ Diabetes also increases the risk of HF through other mechanisms.^12^

Cardiometabolic diseases extend beyond the cardiovascular system, implicating multiple organs. Skeletal muscle, a critical regulator of glucose metabolism, plays a pivotal role in systemic insulin resistance and is directly linked to MetS.^13^ Adipose tissue, particularly visceral fat, contributes to metabolic and proinflammatory abnormalities^14^; in particular, epicardial adipose tissue characteristics are independent risk factors for CAD, atrial fibrillation, and HF.^15–17^

The hematopoietic system is another key player in the pathogenesis of cardiometabolic diseases. Activation of bone marrow (BM) by MetS has been shown to increase the risk of systemic atherosclerosis,^18^ and both the BM and spleen exhibit increased metabolic activity following acute vascular events, such as myocardial infarction or stroke.^19,20^ In addition, MetS affects the brain, as recent evidence links it to neurodegenerative disorders, including Alzheimer disease.^21^

Abdominal organs also play a significant role in cardiometabolic diseases. The interplay between metabolic, cardiovascular, and renal abnormalities, commonly referred to as the cardiovascular-kidney-MetS,^22^ increases the risk of chronic kidney disease, which, in turn, exacerbates vascular events, HF, and cardiovascular mortality.^23^ The liver is closely linked to cardiometabolic abnormalities, as metabolic dysfunction–associated fatty liver disease (MAFLD) is strongly associated with features of the MetS, suggesting shared underlying mechanisms.^24^ Furthermore, pancreatic dysfunction is also associated with cardiometabolic diseases.^25^

Beyond the isolated involvement of individual organs, interorgan crosstalk amplifies and sustains the progression of cardiometabolic diseases. Crucial interactions between systems are essential for maintaining homeostasis, and their disruption leads to increased morbidity.^2^

In this regard, acute conditions can disrupt steady-state interorgan communication, exacerbating systemic responses. The immune system, along with its governing organs, plays a central role in mediating these interactions.^26^ Beyond acute cardiovascular events, other acute conditions, such as cancer onset and its treatments, also induce cardiometabolic alterations.

Multiple methodologies are utilized to assess the impact of cardiometabolic dysregulation both clinically and for research purposes. Among these, imaging technologies play an important role in the phenotypic characterization of metabolic disorders including their early subclinical abnormalities and late clinical manifestations.

In this review, we will explore the pathophysiological insights and the clinical applications that imaging techniques can provide in the evaluation of cardiometabolic diseases, focusing on organ-specific assessments with echocardiography, magnetic resonance imaging (MRI), magnetic resonance spectroscopy (MRS), computed tomography (CT), and positron emission tomography (PET), as well as their combined applications. In addition, we will discuss imaging approaches for evaluating interactions and multiorgan involvement in the pathogenesis of cardiometabolic diseases. Finally, we will examine the role of imaging in detecting disruptions in interorgan communication during acute conditions.

## Imaging Modalities for Cardiometabolic Disease Assessment

### Organ-Specific Imaging Techniques

#### Myocardium

Echocardiography, cardiac CT, cardiac MRI and MRS, and PET imaging can provide insights into the biological mechanisms underlying myocardial diseases. Also, imaging markers can be used as surrogate end points for clinical studies.

##### Imaging Insights of Pathophysiological Mechanisms in Myocardial Metabolism

Advanced imaging techniques have allowed a better understanding of the pathophysiological mechanisms underlying changes in myocardial metabolism. In particular, cardiac MRS is a unique noninvasive technique for assessing cardiac metabolism. While conventional cardiac magnetic resonance (CMR) focuses on water and fat signals (hydrogen nuclei, ^1^H), other nuclei, such as phosphorus (^31^P) and carbon (^13^C), are used for advanced metabolic assessment.

**Figure 1.**
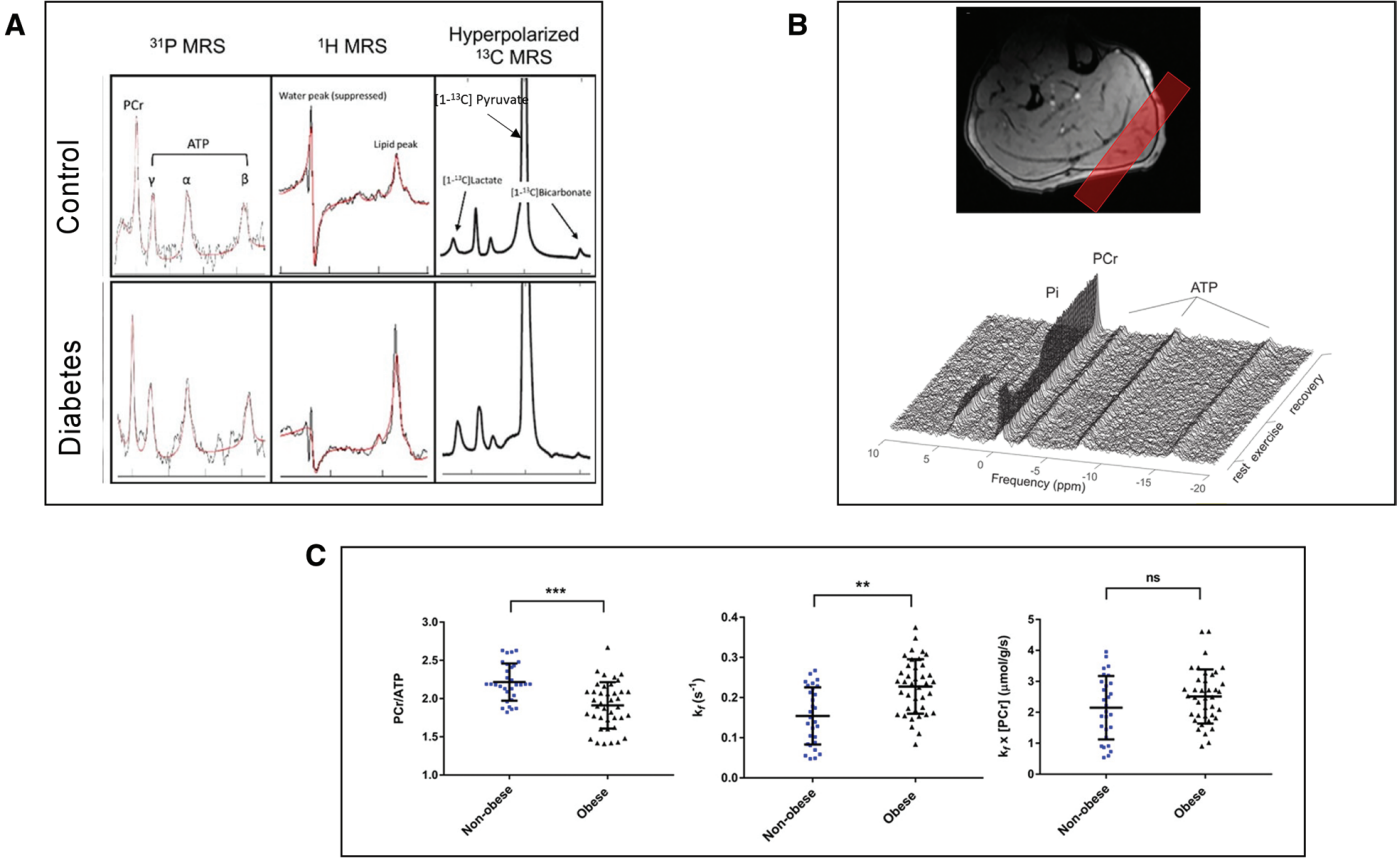
**Magnetic resonance spectroscopy (MRS). A**, Cardiac MRS ^31^P-, ^1^H-, and ^13^C-MRS findings in a patient with diabetes (lower row) compared with healthy control (upper row). Diabetes is associated with impaired myocardial energetics (reduced phosphocreatine/ATP ratio), increased myocardial triglyceride content, elevated lactate-to-pyruvate ratio, and decreased bicarbonate-to-lactate ratio. Adapted from Rider et al^30^ with permission. **B**, Muscle MRS. The upper figure displays noncontrast ^31^P-MRS in a 32-year-old healthy male showing the voxel of interest (red box) positioned on the target muscle (the gastrocnemius). The lower figure presents a graph depicting dynamic spectral data recorded during rest, exercise, and recovery, with peaks corresponding to inorganic phosphate (Pi) and ATP. Adapted from Finnigan et al^31^ with permission. **C**, Cardiac ^31^P-MRS in patients with obesity and without obesity. Obesity is associated with lower phosphocreatine/ATP values, increased forward rate constant of the CK (creatine kinase) creation, and a trend toward increased CK flux. Adapted from Rayner et al^29^ with permission.

^31^P-MRS specifically monitors cardiac high-energy phosphate metabolism; the phosphocreatine/ATP ratio is a common marker of cardiac energy production efficiency, with reductions indicative of metabolic impairment.^22^ In obesity and diabetes, a reduced phosphocreatine/ATP ratio correlates with diastolic dysfunction,^27–29^ which provides a better understanding of the pathophysiological mechanism underlying diabetic cardiomyopathy. Obesity may increase the ATP transfer rate via CK (creatine kinase) as a compensatory mechanism at rest but limits energy reserves during stress,^29^ which explains the higher risk of HF in these patients (Figure 1). In patients with previous myocardial infarction, ^31^P-MRS studies demonstrate reduced phosphocreatine and ATP concentrations.^32^

Hyperpolarized ^13^C-MRS studies have demonstrated that the diabetic myocardium shows reduced pyruvate dehydrogenase flux and increased lactate production, alongside the energetic impairments and lipid accumulation identified by ^31^P- and ^1^H-MRS^30^ (Figure 1). These findings underscore significant metabolic inflexibility, particularly in the heart’s ability to adapt fuel utilization during fasting-to-fed transitions.

Sodium-23 (^23^Na)-MRI also provides information on the myocardial scar and has been used to identify viable myocardium. ^23^Na signal increases in the presence of myocardial ischemia or nonviable scar.^33^

**Figure 2.**
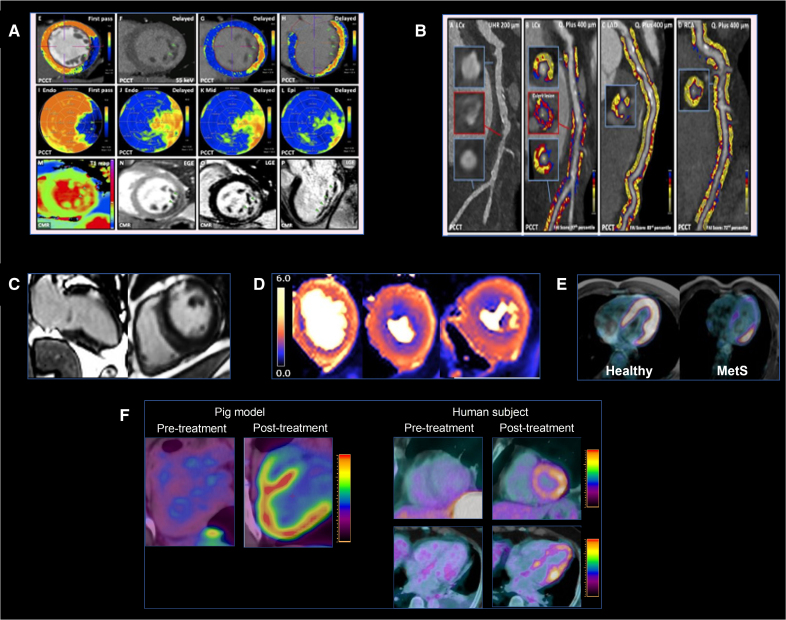
**Myocardial imaging. A**, Myocardial tissue characterization on photon counting computed tomography (PCCT) demonstrating the sensitivity of the technique to myocardial necrosis with cross-validation from cardiac magnetic resonance (CMR) late gadolinium imaging. Adapted from Kotronias et al^34^ with permission. **B**, PCCT images from a patient with an inferolateral myocardial infarction reveal increased vascular fat attenuation index (FAI) in the LCx (left circumflex coronary artery) and relatively normal FAI in the other vessels. Adapted from Kotronias et al^34^ with permission. **C**, Late gadolinium enhanced imaging of a patient with diabetes, showing anterior subendocardial myocardial infarction (red arrows). **D**, Pixelwise perfusion images from a hypertensive patient with metabolic syndrome, demonstrating inducible perfusion defect in the anterior wall (black arrow), suggestive of microvascular dysfunction. **E**, ^18^F-FDG (^18^F-fluorodeoxyglucose) positron emission tomography/ magnetic resonance imaging comparing a healthy individual (**left**) with diffuse physiological myocardial ^18^F-FDG uptake and a patient with metabolic syndrome (**right**) with reduced myocardial ^18^F-FDG uptake. Images were obtained from the PESA study (Progression of Early Subclinical Atherosclerosis). **F**, Cardiac metabolic alterations following anthracycline-based therapy. The figure illustrates increased ^18^F-FDG uptake in the myocardium observed in both a pig model and human subjects after anthracycline treatment, indicating a metabolic shift toward glucose utilization. Images were obtained from a project funded by the European Research Council under the European Union’s Horizon 2020 Research and Innovation Program (grant agreement 819775).

^18^F-FDG (^18^F-fluorodeoxyglucose) PET is another essential tool for the study of myocardial metabolism. Previous studies have demonstrated that myocardial metabolism is impaired in the context of MetS and insulin resistance, even in asymptomatic individuals who seem otherwise healthy^11^ (Figure 2). These individuals exhibit reduced ^18^F-FDG uptake in the myocardium, indicating decreased glucose consumption. Similar reductions in myocardial glucose uptake are observed in patients with diabetes,^35^ and this metabolic abnormality is associated with an elevated risk for atherosclerotic disease.^11^ The shift in myocardial energetic substrate utilization also correlates with reduced myocardial efficiency,^36^ which has been linked to the development of HF and adverse cardiovascular events.^37^ Furthermore, ^18^F-FDG PET imaging has revealed that anthracycline-based cancer treatments induce a marked metabolic shift in the heart, favoring glucose over fatty acids as the primary substrate for energy production^38^ (Figure 2), which, in turn, may have consequences on cardiac efficiency and atherosclerotic risk as described above.

Novel radiotracers help in identifying early changes occurring in the myocardium, such as fibrosis and inflammation. For instance, ^68^Ga-fibroblast activation protein inhibitor PET allows early identification of myocardial fibrosis.^39^ ^68^Ga-DOTATATE is used to detect inflammation associated with myocardial infarction.^40^ These techniques allow a better understanding of early changes associated with cardiometabolic diseases.

##### Clinical Applications of Cardiac Imaging

Echocardiography is the first-line imaging modality in clinical practice for assessing the cardiac impact of cardiometabolic diseases. Studies using echocardiography show that left ventricular (LV) concentric remodeling is associated with insulin resistance, diabetes, hyperleptinemia, myocardial steatosis, and visceral adipose tissue expansion.^41^ In obese individuals, LV dilatation is frequently observed, driven by increased fat mass, elevated cardiac output, and greater total blood volume.^42^ Increased central adiposity and weight gain contribute to LV diastolic stiffness, impairing diastolic function.^43^ While LV ejection fraction may remain within normal ranges for extended periods, studies examining myocardial deformation and active shortening and thickening have revealed impaired LV strain in longitudinal, circumferential, and radial directions.^44^

Cardiac CT provides a comprehensive evaluation of cardiometabolic health by assessing coronary arteries,^41^ fat distribution, and other metabolic health markers (Figure 2). Coronary artery calcium scoring allows for the evaluation of calcified plaques, which correlate with atherosclerotic burden and serve as a surrogate marker for cardiovascular risk. CT studies have consistently demonstrated a high prevalence of coronary stenosis, multivessel involvement, and an increased total coronary plaque burden in individuals with diabetes and other metabolic conditions. In particular, atherosclerotic plaques in these populations often exhibit higher lipid content and lower fibrotic composition, making them more vulnerable to rupture.^45,46^ PET techniques such as ^18^F-sodium fluoride allow the identification of vascular microcalcifications related to atherosclerosis,^47^ providing another dimension to CAD evaluation. CT also enables the evaluation of extracellular volume, with CT-derived extracellular volume measurements showing excellent correlation with those obtained by CMR, the gold standard technique.^48^ Moreover, CT-derived extracellular volume is a recognized risk marker for HF, ventricular arrhythmias, and mortality.^49^ CT also provides precise measurements of perivascular fat, which is discussed further in a later section. In addition, CT enables the monitoring of therapeutic interventions, providing insights into treatment efficacy over time.^50,51^

**Figure 3.**
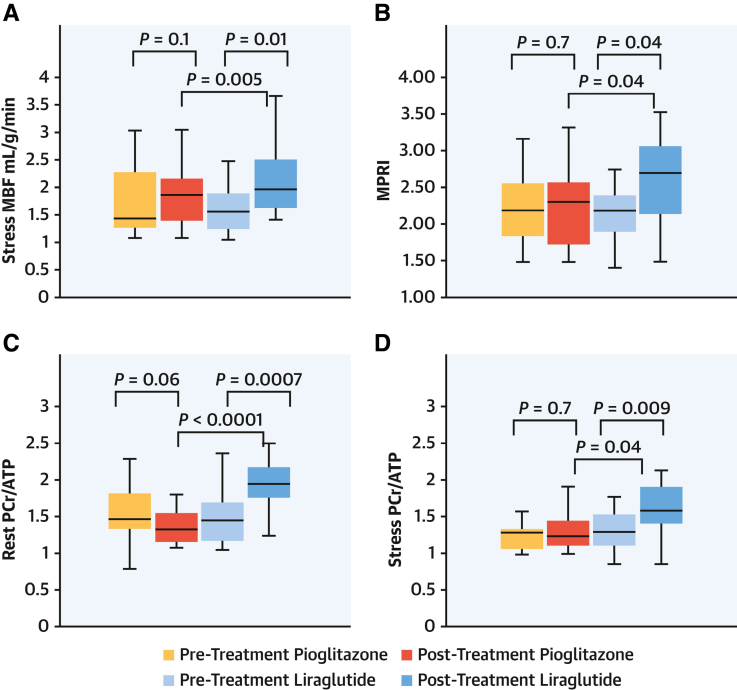
**Role of imaging techniques in monitoring treatment outcomes for cardiometabolic diseases.** Stress cardiac magnetic resonance imaging (stress myocardial blood flow [MBF; **A**] and myocardial perfusion reserve index [**B**]) and ^31^P-magnetic resonance spectroscopy (**C** and **D**) for monitoring the effects of liraglutide and pioglitazone in patients with diabetes. The graphs demonstrate that liraglutide improves myocardial perfusion and energy metabolism in patients with type 2 diabetes. Reproduced from Chowdhary et al^57^ with permission. MPRI indicates myocardial perfusion reserve index; and PCr, phosphocreatine.

CMR studies have demonstrated subtle LV hypertrophy^52^ and abnormalities in both systolic and diastolic functions,^53^ detectable through cine imaging and myocardial and atrial systolic and diastolic deformation or strain analysis. CMR can quantify extracellular matrix alterations and myocardial fibrosis using T1 mapping and extracellular volume quantification,^54^ which may be elevated in diabetic cardiomyopathy and is also linked to poor outcomes (Figure 2). Stress T1 mapping, a measure of blood volume reserve, is reduced in patients with diabetes, indicating coronary microvascular dysfunction.^55^ In addition, in both early diabetes and obesity, myocardial blood flow (MBF) and myocardial perfusion reserve^56^ as assessed on dynamic contrast-enhanced (DCE) first-pass perfusion CMR may be impaired as discussed in a later section. Many of these techniques have been applied to track response to novel therapies (eg, empagliflozin and liraglutide) for diabetes and cardiometabolic diseases^57,58^ (Figure 3). Late gadolinium imaging is also invaluable in the assessment of vascular complications of MetS including the detection of myocardial infarction and associated complications (eg, aneurysms and ventricular rupture) and assessment of myocardial viability. Multiparametric MRI of the postischemic heart identifies a severe inflammatory response with a bimodal pattern: an early wave of edema linked to reactive hyperemia and increased vascular permeability, followed days later by a second wave due to inflammatory cell infiltration originating from the BM and spleen.^59^

Cardiac MRS is also useful in the monitoring of therapeutic interventions. Intentional weight loss and specific treatments, such as SGLT2 (sodium-glucose cotransporter 2) inhibitors and GLP-1 (glucagon-like peptide 1) receptor agonists, have been shown to improve phosphocreatine/ATP ratios and cardiac function as evaluated by ^31^P-MRS.^57,60,61^ ^1^H-MRS detects intramyocardial lipid levels that are elevated in obesity and diabetes, correlating with cardiac remodeling and dysfunction.^62^ Furthermore, results from dietary intervention studies have shown that myocardial triglyceride content is modifiable and related to cardiac function in obesity and diabetes.^63^ Furthermore, given the intertwined relationship between diabetes, obesity, and HF, the ability to measure cardiac metabolism will become increasingly important in tailoring interventions to individual patients and ensuring optimal patient outcomes.

#### Coronary Microcirculation

Imaging myocardial microcirculation is critical for understanding myocardial perfusion and identifying early vascular changes associated with cardiometabolic diseases. Techniques such as myocardial contrast echocardiography, CMR, and PET are central to these evaluations.

##### Imaging Insights of Pathophysiological Mechanisms in Microvascular Dysfunction

Advanced imaging techniques provide deeper insights into the early and subtle changes occurring in coronary microcirculation, often long before clinical disease becomes apparent. The use of quantitative perfusion techniques in DCE-MRI has enabled the identification of early alterations in microvascular function in asymptomatic adults.^7^ In one study, resting MBF was directly correlated with the number of MetS components, as well as with insulin resistance, assessed using the homeostatic model assessment for insulin resistance and the presence of diabetes.^7^ These findings align with those of a previous study in patients with diabetes^8^ and suggest that in cardiometabolic diseases, not only stress MBF is reduced, but also resting MBF is increased, likely due to altered basal endothelial mechanisms. This imbalance contributes to a decrease in myocardial perfusion reserve. Such changes are only detectable through quantitative techniques, which offer high sensitivity for detecting subtle perfusion alterations.

While DCE-MRI is a well-established technique, emerging methods such as arterial spin labeling, oxygen-sensitive or blood-oxygen-level-dependent MRI,^64^ and intravoxel incoherent motion also show promise in identifying the pathophysiological mechanisms underlying alterations in coronary microcirculation and myocardial oxygen reserve without the need for contrast agents.

##### Clinical Applications of Microvascular Imaging

In clinical practice, myocardial contrast echocardiography is particularly useful for identifying localized microvascular abnormalities, and it has shown that MBF reserve is reduced in patients with diabetes with no CAD.^65^

The high sensitivity of DCE-MRI to perfusion changes makes it particularly suitable for diagnosing coronary microvascular dysfunction, which is increasingly recognized as a contributor to ischemic heart disease, obesity, and diabetic cardiomyopathy (Figure 2).^66^ In diabetic cardiomyopathy, DCE-MRI is particularly valuable for detecting diffuse perfusion defects, serving as early indicators of cardiovascular risk, and guiding therapeutic interventions to improve outcomes.^8,67^ In addition, DCE-MRI is valuable for assessing other conditions, such as anthracycline-induced cardiotoxicity where irreversible cardiac microcirculatory dysfunction may develop.^68^

PET-determined myocardial perfusion also offers an effective method for assessing coronary microcirculation. Research indicates that coronary microvascular dysfunction as assessed by PET is notably prevalent among individuals with diabetes, MetS, and obesity.^69^ Furthermore, a recent large-scale study has highlighted that abnormal PET findings can reliably identify patients at high risk of cardiometabolic disease, providing critical diagnostic and prognostic insights.^70^

#### Arterial System

The arterial system plays a central role in the development, progression, and consequences of cardiometabolic diseases.^71^ Cardiometabolic diseases often induce structural and functional changes in the arterial system, contributing to the formation of atherosclerosis,^72^ which can result in cardiovascular complications.

##### Imaging Insights of Pathophysiological Mechanisms in Atherosclerosis

Multimodality imaging, such as ^18^F-FDG PET/CT and PET/MRI, offers detailed information on the activity and composition of atherosclerotic plaques.^73^ Importantly, ^18^F-FDG PET enables the detection of early changes in the vascular wall, even before overt atherosclerotic plaques are identifiable^74^; this information has been crucial in advancing our understanding of the inflammatory processes underlying atherosclerosis formation. Moreover, PET studies have demonstrated that arterial wall inflammation is directly influenced by MetS^75,76^ and diabetes,^77^ highlighting their contribution to the atherosclerotic process.

##### Clinical Applications of Vascular Imaging

Vascular ultrasound enables the evaluation of atherosclerotic plaque and arterial stiffness,^78–80^ a technique highlighted in the latest ESC guidelines as a key tool for cardiovascular risk evaluation.^81^ Furthermore, the progression of atherosclerosis, as evaluated by vascular ultrasound, has been shown to be an independent predictor of all-cause mortality^.82^ Thus, this noninvasive and cost-effective tool provides valuable prognostic information.

MRI can also be used to study vascular compliance^83^ and aortic stiffness, which are abnormal in cardiometabolic diseases^84^ and an independent predictor of vascular outcomes. In addition, MRI can further characterize atherosclerotic plaque composition and identify features of vulnerability, which can predict the risk for vascular events.^85^ Extracardiac vascular imaging with CT allows the evaluation of vascular calcification in the aorta, renal arteries, and peripheral arteries, which has been associated with cardiometabolic risk and correlates with overall atherosclerotic burden.^86^

#### Skeletal Muscle

Skeletal muscle dysfunction is a hallmark of cardiometabolic diseases, characterized by metabolic inflexibility, or the impaired ability of muscles to switch between lipid and carbohydrate oxidation depending on fuel availability.^87^ This dysfunction results in the accumulation of ectopic lipids within both intramyocellular and extramyocellular compartments of the muscle, disrupting normal metabolism.

##### Imaging Insights of Pathophysiological Mechanisms in Muscle Dysfunction

^1^H-MRS is considered the gold standard for differentiating between intramyocellular and extracellular lipids.^88^ In addition, the Dixon MRI method enables the spatial visualization of fat distribution within the skeletal muscle.^89^ Studies combining Dixon MRI with ^1^H-MRS have demonstrated elevated intramyocellular lipid levels in patients with cardiometabolic diseases, correlating with measures of insulin sensitivity.^90^

^31^P-MRS can also provide insights into skeletal muscle bioenergetics (Figure 1). Static and dynamic ^31^P-MRS imaging has revealed that patients with cardiometabolic diseases exhibit delayed phosphocreatine recovery, altered inorganic phosphorus-to-ATP exchange flux, and changes in glycerophosphocholine concentrations, indicative of impaired mitochondrial ATP production and oxidative capacity.^91,92^ These findings explain the impact of cardiometabolic diseases on skeletal muscle metabolism.^93^

^18^F-FDG PET has also been used to study the effects of cardiometabolic diseases on skeletal muscle. Studies have shown reduced ^18^F-FDG uptake in the skeletal muscle of patients with diabetes, indicating impaired glucose utilization. This reduction is closely associated with systemic insulin resistance.^94^

##### Clinical Applications of Skeletal Muscle Imaging

Muscle quantity and quality are important markers of cardiometabolic risk. Sarcopenia has been associated with insulin resistance and increased risk of cardiovascular disease.^95^ In clinical practice, CT and MRI are the reference techniques for assessing sarcopenia and evaluating cardiometabolic risk. CT studies show that muscles with normal density exhibit attenuation values around 31 to 100 Hounsfield unit (HU), while fat-infiltrated muscles present lower values, typically ranging from 0 to 30 HU.^96^ One CT study demonstrated that decreased muscle mass is an independent predictor of cardiovascular events and HF.^97^ These findings suggest that muscle mass and composition could serve as valuable markers for monitoring treatment response in cardiometabolic diseases.

Dixon MRI also provides an accurate quantification of muscle fat content.^89^ In addition, muscle function is linked to cardiovascular risk.^98^ ^31^P-MRS allows for the evaluation of muscle function^31^; although not yet widely available for clinical practice, this technique holds promise for assessing cardiometabolic risk.

#### Adipose Tissue

Imaging of visceral, subcutaneous, and perivascular fat provides critical insights into obesity, diabetes, and MetS, where adipose tissue distribution serves not only as a marker of disease severity but also as a crucial factor in disease progression.

##### Imaging Insights of Pathophysiological Implications of Adipose Tissue Inflammation

**Figure 4.**
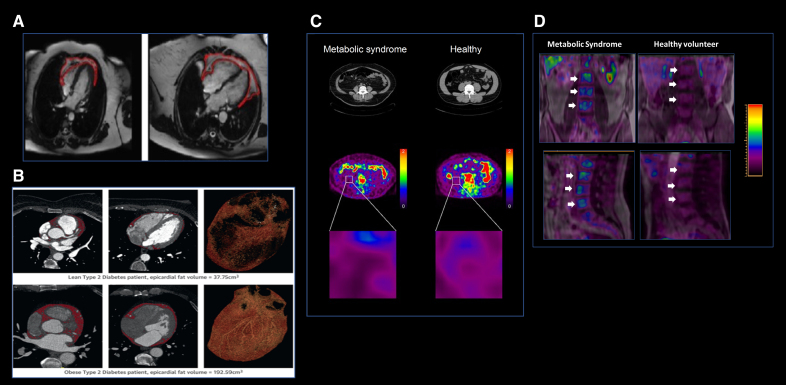
**Adipose tissue and bone marrow imaging. A**, Example of magnetic resonance (MR) images showing automated periadipose tissue segmentation and predicted segmentation quality. Adapted from Ardissino et al^104^ with permission. **B**, Cardiac computed tomography (CT) images of epicardial fat segmentation from a patient with diabetes. The lower row shows increased epicardial fat volume in a patient with obesity and diabetes compared with the lean patient in the upper row. Adapted from Levelt et al^144^ with permission. **C**, ^18^F-FDG (^18^F-fluorodeoxyglucose) positron emission tomography (PET)/CT images of visceral adipose tissue (VAT) and subcutaneous adipose tissue in a patient with metabolic syndrome (**left**) and a healthy individual (**right**). ^18^F-FDG uptake in VAT is notably higher in patients with metabolic syndrome compared with healthy individuals. Adapted from Pahk et al^103^ with permission. **D**, ^18^F-FDG PET/MR imaging of the bone marrow in metabolic syndrome and healthy individuals. The upper figure demonstrates increased ^18^F-FDG uptake in the lumbar vertebrae (white arrows) of a patient with metabolic syndrome, whereas the lower figure shows lower uptake in a healthy individual. Images were obtained from the PESA study (Progression of Early Subclinical Atherosclerosis).

Adipose tissue inflammation has been linked to cardiometabolic diseases, an association primarily studied in patients with obesity.^99^ In fact, it has been suggested that metabolic diseases may progress due to persistent inflammation in key cardiometabolic tissues, including adipose tissue.^100^ Experimental studies using in vivo imaging have demonstrated activation of the leukocyte adhesion cascade in the adipose tissue of obese mice, indicating an active inflammatory process.^101^ In humans, ^18^F-FDG PET is the most frequently used imaging technique for assessing adipose tissue inflammation. ^18^F-FDG uptake in adipose tissue has been associated with insulin resistance and adiponectin levels,^102^ reflecting the inflammatory status in MetS^103^ (Figure 4). In addition, increased ^18^F-FDG uptake in adipose tissue has been linked to inflammation in other cardiometabolic organs, highlighting the connection between adipose tissue inflammation and systemic insulin resistance. For instance, adipose tissue ^18^F-FDG uptake is associated with vascular inflammation^93^ and has been associated with reduced cerebral glucose metabolism, suggesting a connection between adipose tissue dysfunction and neurodegenerative diseases.^105^

Other radiotracers, such as ^18^F-fluoro-6-thia-heptadecanoic acid and ^11^C-acetate, have been suggested to provide a more accurate assessment of adipose tissue metabolic activity compared with ^18^F-FDG.^106^

A particular type of fat, the perivascular adipose tissue (PVAT), has been recognized as a dynamic endocrine organ that plays a crucial role in vascular function. CT imaging of PVAT has demonstrated that fat density is closely related to vascular inflammation, suggesting that PVAT composition may serve as a biomarker for atherosclerosis.^107^ The fat attenuation index (FAI), a CT-derived quantitative metric of PVAT density, has been shown to provide critical insights into vascular inflammation associated with cardiometabolic disease. FAI reflects the HU density of PVAT, which is higher when PVAT is inflamed (Figure 2).

##### Clinical Applications of Fat Imaging

Visceral fat, which is strongly associated with insulin resistance and inflammation, can be quantified using specific MRI sequences such as proton density fat fraction,^108^ T2, T2*, or Dixon imaging,^109^ or by CT through the use of HU.^110^ Although subcutaneous fat is less pathogenic, its measurement alongside visceral fat helps refine assessments of total adiposity and its varied metabolic impacts. In fact, the volume and density of visceral and subcutaneous fat correlate with cardiovascular risk and can serve as predictors for metabolic outcomes.^111^ In addition, both CT and MRI may also provide valuable insights into adipose tissue inflammation, which may be inferred from altered HU values in CT or higher T2 values in MRI (Figure 4). Indeed, lower fat attenuation values have been associated with worse cardiometabolic profiles at follow-up.^111^

PVAT has also been shown to have relevant clinical implications. Recent studies highlight FAI’s prognostic value in predicting cardiovascular events. Elevated FAI was independently linked to myocardial infarction and adverse plaque features. The ORFAN (Oxford Risk Factors And Non-invasive imaging) study^112^ investigators have further confirmed its role in risk stratification, especially in nonobstructive CAD, by combining FAI with plaque metrics. Advances in artificial intelligence have enhanced FAI sensitivity, enabling the detection of even subtle inflammatory PVAT changes that correlate with systemic inflammatory markers. In MetS and diabetes, increased FAI reflects poorer metabolic control and cardiovascular risk, independent of obesity metrics.^107^ FAI also allows tracking of treatment effects (eg, statin use), highlighting its role in personalized risk stratification and management strategies of cardiometabolic diseases.^107,113^

Epicardial adipose tissue is an important risk marker in cardiometabolic diseases. In pathological states such as MetS and other inflammatory disorders, epicardial adipose tissue undergoes expansion and functional alterations, leading to a proinflammatory profile.^114^ Its volume can be quantified using cardiac CT and MRI,^15,17^ with CT offering additional insights. Specifically, epicardial adipose tissue inflammation can be detected through increased epicardial adipose tissue attenuation on CT scans, characterized by higher HU values; this feature is linked to cardiovascular disease^115^ (Figure 4).

#### Bone Marrow

##### Imaging Insights of Pathophysiological Mechanisms in BM Metabolic Activity

Experimental studies have suggested that the BM plays a significant role in cardiometabolic diseases. Cardiometabolic risk factors increase the release of hematopoietic progenitor cells, which impact systemic inflammation and accelerate atherosclerosis progression.^116^ In humans, the study of BM can only be performed using hybrid imaging methods that combine both anatomic and metabolic information. One study in humans used hybrid PET/MRI techniques to quantify BM activity^18^ (Figure 4). This research demonstrated that BM activation, as evidenced by increased ^18^F-FDG uptake in the vertebrae of asymptomatic individuals, is associated with MetS and its individual components, as well as with systemic inflammation and elevated leukocyte counts.^18^ In addition, as proposed by preclinical studies, BM activation is linked to early signs of atherosclerosis (ie, increased ^18^F-FDG vascular uptake^74^). The concurrent increase in ^18^F-FDG uptake in both the BM and arteries is associated with a higher prevalence of atherosclerosis and a greater plaque burden.^18^

Acute cardiovascular complications of MetS are also known to significantly impact BM metabolism. Studies using ^18^F-FDG PET have shown that BM activity increases in patients experiencing acute cardiovascular events.^19,20^ Myocardial infarction or stroke activates the BM, stimulating the proliferation of hematopoietic stem and progenitor cells. This process leads to increased production of neutrophils and inflammatory monocytes, which contribute to poor myocardial healing.^117^ Moreover, BM activation following myocardial infarction contributes to atherosclerosis progression.^116^ Interestingly, patients with stable cardiovascular disease also exhibit higher BM metabolic activity.^118^

##### Clinical Application of BM Imaging

Experimental studies have proposed imaging methods for evaluating BM activity as potential markers for treatment monitoring. In one study^119^ in mice, PET/MRI enabled the detection of increased BM vasculature following exposure to a danger signal. Clinical studies in inflammatory diseases, such as rheumatoid arthritis, have shown that BM edema evaluated by MRI can identify inflammatory infiltrates in these patients.^120^ Altogether, these studies suggest that BM activity, evaluated through noninvasive advanced imaging techniques, could serve as a valuable tool for monitoring the efficacy of therapies with anti-inflammatory effects.

#### Brain

##### Imaging Insights of Pathophysiological Mechanisms in Neurodegenerative Diseases

**Figure 5.**
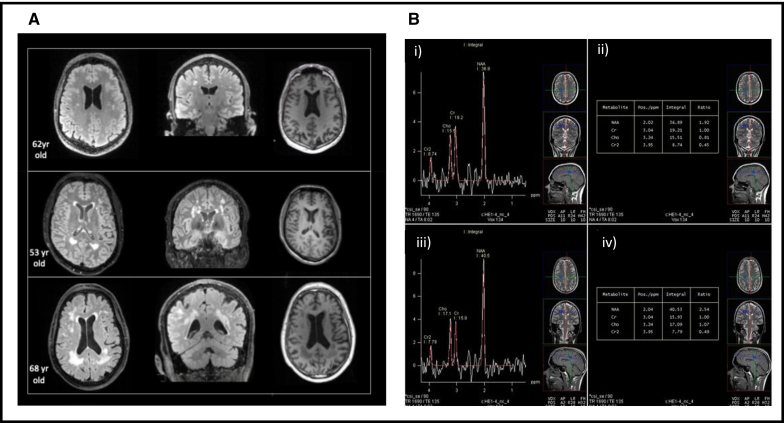
**Brain magnetic resonance imaging (MRI) and magnetic resonance spectroscopy (MRS). A**, Brain MRI showing hyperintensities on T2 FLAIR (Fluid Attenuated Inversion Recovery) and T1-weighted imaging in asymptomatic individuals with cardiometabolic risk factors, suggestive of subclinical vascular injury. **B**, Long-echo multivoxel MRS in the region of the right deep frontal white matter. Spectrum (i) in an obese subject, showing a decrease in the N-acetylaspartate (NAA)/creatine (Cr) and choline-containing molecules (Cho)/Cr ratio (ii); spectrum (iii) in a control subject, showing a normal ratio of NAA/Cr and Cho/Cr. Adapted from Vuković et al^124^ with permission.

The breakdown of the blood-brain barrier has been observed in preclinical models of obesity and diabetes, which is thought to exacerbate neuroinflammation and brain injury.^121^ MRI techniques such as DCE-MRI, diffusion-weighted imaging, tensor imaging, and susceptibility-weighted imaging hold the potential for characterizing the blood-brain barrier in cardiometabolic diseases.^122,123^ Complementing these structural imaging methods, a recent study utilizing cerebral MRS revealed that central obesity and hyperinsulinemia are associated with neurometabolic alterations, including reduced N-acetylaspartate and choline levels in brain regions involved in cognitive and emotional processing^124^ (Figure 5).

^18^F-FDG PET studies have also shown that cardiometabolic diseases alter brain glucose uptake, influenced by both local mechanisms and systemic glucose levels.^125,126^ In asymptomatic middle-aged individuals, cardiometabolic risk factors, especially hypertension, are associated with areas of brain hypometabolism.^127^ Regional blood flow, as assessed by PET, may also be altered in obese participants.^128^ In patients with insulin resistance, ^18^F-FDG PET reveals abnormal glucose uptake in brain regions susceptible to neurodegeneration although the results in this are mixed.^94,129,130^

Imaging tracers targeting neuroinflammatory markers (eg, TSPO [translocator protein], COX-2 [cyclooxygenase-2], CB2 [cannabinoid receptor 2], MAO-B [monoamine oxidase-B], and P2X7 [purinergic receptor P2X 7]) have been used to study brain inflammation, with mixed results in MetS models. This could be due to less robust selectivity for microglial activation, a feature that is still under investigation.^131^ Furthermore, studies have shown that ^18^F-florbetaben PET imaging can detect amyloid plaques in individuals with diabetes, hypertension, and hypercholesterolemia, diagnosed years before PET, thus linking cardiometabolic diseases with neurodegeneration.^132^

These imaging findings allow a better understanding of the processes underlying cognitive dysfunction in patients with cardiometabolic diseases.

##### Clinical Applications of Brain Imaging

Structural brain changes evaluated by MRI, particularly atrophy, are commonly observed in individuals with obesity and MetS, particularly in regions such as the frontal lobes, anterior cingulate gyrus, hippocampus, amygdala, brainstem, and thalamus. Both gray and white matter losses have been reported in patients with diabetes, and these changes are associated with cognitive decline.^133^ White matter hyperintensities are commonly observed in individuals with MetS, often alongside markers of cerebrovascular disease, which increase the risk of stroke and cognitive impairment^134^ (Figure 5). Finally, functional brain MRI and perfusion studies have shown impaired cerebral perfusion in areas crucial for cognition in patients with diabetes and obesity.^125^

#### Liver

Individuals with cardiometabolic diseases often exhibit liver abnormalities such as MAFLD and metabolic dysfunction–associated steatohepatitis.^135^ Various imaging techniques, particularly MRI, are pivotal in assessing these liver conditions.

##### Imaging Insights of Pathophysiological Mechanisms in MAFLD and Metabolic Dysfunction–Associated Steatohepatitis

^13^C-MRS and ^31^P-MRS^136–138^ have been utilized to assess hepatic oxidative metabolism in cardiometabolic diseases. These studies reveal lower ATP turnover^139^ and slower tricarboxylic acid flux in individuals with diabetes, indicative of impaired liver metabolism. Measures such as liver steatosis, body mass index, waist circumference, fasting glucose, and HbA1c (glycated hemoglobin) also correlate with altered liver metabolism.^139^ Studies using ^18^F-FDG PET have demonstrated increased ^18^F-FDG uptake in the liver of individuals with MetS.^103,140^ These imaging findings further explain the link between liver dysfunction and cardiometabolic syndrome.

##### Clinical Applications of Liver Imaging

**Figure 6.**
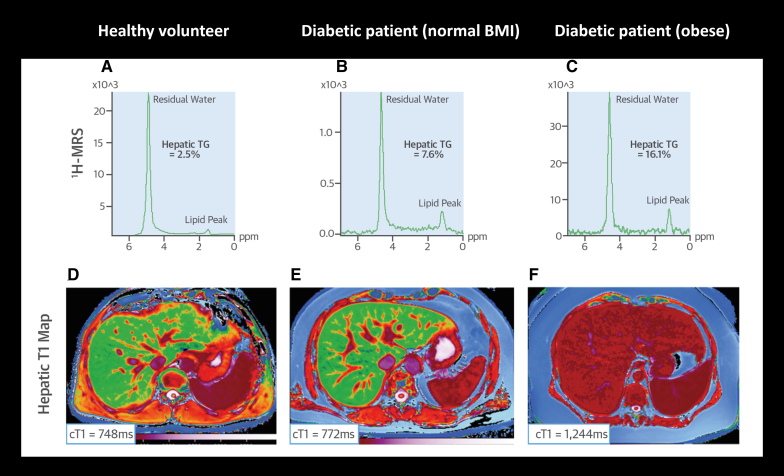
**Liver magnetic resonance spectroscopy (MRS) in patients with diabetes compared with healthy volunteers. A**, ^1^H-MRS of a healthy volunteer with hepatic triglyceride (TG) content of 2.5%. **B**, ^1^H-MRS of a lean patient with diabetes with hepatic TG content of 7.6%. **C**, ^1^H-MRS of a patient with obesity and diabetes with hepatic TG content of 16.1%. **D**, ShMOLLI (Shortened Modified Look-Locker Inversion Recovery) T1 map of the liver in a healthy volunteer showing a corrected T1 (cT1) of 748 ms. **E**, ShMOLLI T1 map of the liver in a lean patient with diabetes with a cT1 of 772 ms. **F**, ShMOLLI T1 map of the liver in a patient with obesity and diabetes with a cT1 of 1244 ms. BMI indicates body mass index. Adapted from Levelt et al^144^ with permission.

Studies^141^ using ultrasound have shown that individuals with cardiometabolic syndrome exhibit greater liver stiffness (a marker of fibrosis) compared with those without, correlating with insulin resistance and systemic inflammation.^142^ Quantitative MRI techniques, including tissue-specific relaxometry mapping, have also proven useful for assessing tissue characteristics such as fibrosis, edema, and iron levels, offering valuable insights into liver pathology and its relationship with cardiometabolic health^143^ (Figure 6). Notably, iron-corrected T1 mapping serves as a surrogate marker for liver inflammation and fibrosis and has been shown to be more prevalent among patients with obesity with diabetes. Higher corrected T1 levels have also been linked to an increased risk of major cardiovascular events such as atrial fibrillation, HF, and all-cause mortality, independent of liver fat content and metabolic health status.^145^ Altered iron homeostasis may also correlate with insulin resistance in obese individuals, highlighting the importance of iron metabolism in the context of cardiometabolic syndromes.^144^

Liver fat content is also a prognostic factor in metabolic diseases. MRI-based proton density fat fraction imaging has demonstrated higher liver fat content in individuals with cardiometabolic diseases compared with healthy individuals without metabolic dysfunction.^1461^H-MRS has further revealed increased liver fat content in individuals with diabetes and obesity, with higher liver fat associated with an increased risk of cardiovascular events in a multiethnic population^147^ (Figure 6).

#### Pancreas

Obesity, inflammation, insulin resistance, sleep apnea, and environmental factors are common mechanisms linking cardiometabolic diseases with pancreatic dysfunction.^25^

##### Imaging Insights of Pathophysiological Mechanisms in Pancreatic Function

Pancreatic MRI and MRS may be used to investigate pancreatic fat content and its association with metabolic dysfunction. A meta-analysis of studies evaluating pancreatic size and fat content through ultrasound, CT, and MRI concluded that excess pancreatic fat seen in diabetes leads to β-cell reduction and impaired insulin secretion, contributing to hyperglycemia and metabolic dysregulation.^148^ Individuals with diabetes and obesity often exhibit increased pancreatic fat, which correlates with factors such as waist circumference, triglyceride levels, hyperferritinemia, visceral adipose tissue, and insulin resistance.^149,150^

Studies using ^31^P- and ^13^C-MRS have shown reduced oxidative metabolism in pancreatic cells in individuals with cardiometabolic dysfunction, thought to underlie β-cell dysfunction and reduced insulin secretion.^151^

PET imaging with tracers targeting GLP-1 receptors such as radiolabeled exendin-4 provides a noninvasive assessment of β-cell mass in the pancreas.^152–154^ Studies in both animals and humans have shown reduced tracer uptake in diabetes and insulin-resistant models, indicating a reduction in β-cell mass.^155^ Single-photon emission CT imaging with indium-111-labeled GLP-1 receptor probes^156^ has also shown decreased β-cell signal in these models, indicating a reduction in β-cell mass^154^ and possibly impaired insulin secretion.

PET perfusion imaging has been used to assess pancreatic blood flow, revealing early abnormalities in obesity, which suggest compromised β-cell function due to inadequate nutrient and oxygen delivery.^157^

##### Clinical Applications of Pancreatic Imaging

MRS techniques have been shown useful in the study of pancreatic fat in patients with obesity. Particularly, in patients undergoing bariatric surgery or short-term caloric restriction, ^1^H-MRS has shown significant reductions in pancreatic fat and improvements in β-cell function, highlighting the modifiable natures of these changes.^158^

Research on GLP-1 agonists has shown that these therapies can improve imaging signals of β-cell function,^159^ underscoring the potential reversibility of pancreatic dysfunction with targeted cardiometabolic therapies.

#### Kidneys

##### Imaging Insights of Pathophysiological Mechanisms in Renal Diseases

Nuclear imaging modalities have shown promise in assessing renal pathophysiology. Techniques such as technetium-99 m-diethylenetriaminepentaacetic acid and technetium-99 m-mercaptoacetyltriglycine PET can evaluate renal perfusion, while ^18^F-FDG and ^68^Ga-fibroblast activation protein inhibitor PET offer insights into renal inflammation and fibrosis.^160–163^

##### Clinical Applications of Renal Imaging

Renal MRI is increasingly used in the assessment of patients with MetS, offering precise evaluation of kidney size, corticomedullary differentiation, and early kidney damage. Advanced techniques such as T1 and T2 mappings allow for the detection of renal fibrosis and inflammation, while renal fat fraction quantification provides insights into progressive renal dysfunction, particularly in diabetes.^164^ Furthermore, increased renal fat content, as detected on MRI, has been linked to obesity-related renal injury.^165^ Noncontrast MRI approaches, including arterial spin labeling and oxygen-sensitive or blood-oxygen-level-dependent imaging, enable the assessment of renal perfusion and oxygenation. These methods are particularly valuable for identifying abnormalities in renal function before the onset of overt nephropathy. Oxygen-sensitive or blood-oxygen-level-dependent imaging, for instance, can noninvasively detect renal hypoxia in diabetes, with studies showing more pronounced hypoxia in the medulla compared with the cortex.^166^ Similarly, arterial spin labeling has demonstrated the ability to quantify early renal perfusion impairment in diabetes, even before changes in glomerular filtration rate are evident. Functional MRI techniques, such as diffusion-weighted imaging, are emerging as effective tools for evaluating tubular function and tissue integrity. DCE-MRI also provides novel methods to estimate renal filtration.

#### Spleen

##### Imaging Insights of Pathophysiological Mechanisms in Splenic Metabolism

Splenic activity can be assessed on imaging modalities such as ^18^F-FDG PET. Increased ^18^F-FDG uptake may indicate heightened immune activation in the context of MetS; however, this area deserves further investigation. Emerging tracers, such as ^68^Ga-DOTATATE and TSPO tracers, may offer more specific insights into inflammation and immune activity in the spleen, enhancing our understanding of splenic involvement in cardiometabolic conditions.^167,168^

##### Clinical Applications of Splenic Imaging

Noninvasive imaging of the spleen provides unique insights into its structure and function in the context of MetS, obesity, and diabetes, as these conditions are associated with systemic inflammation and immune dysregulation. Abdominal MRI can accurately measure splenic size and volume, which have been found to increase in individuals with obesity. Advanced techniques such as T1 and T2 mappings are sensitive to changes in splenic vascularity and fibrosis, while T1 mapping may also be used to detect alterations in iron homeostasis within the spleen.^169^

### Contributions of Each Imaging Technique to the Evaluation of Cardiometabolic Diseases

The different imaging techniques can contribute to the evaluation of cardiometabolic diseases in several ways. The Table represents the main uses of each technique in cardiometabolic diseases.

**Table.**
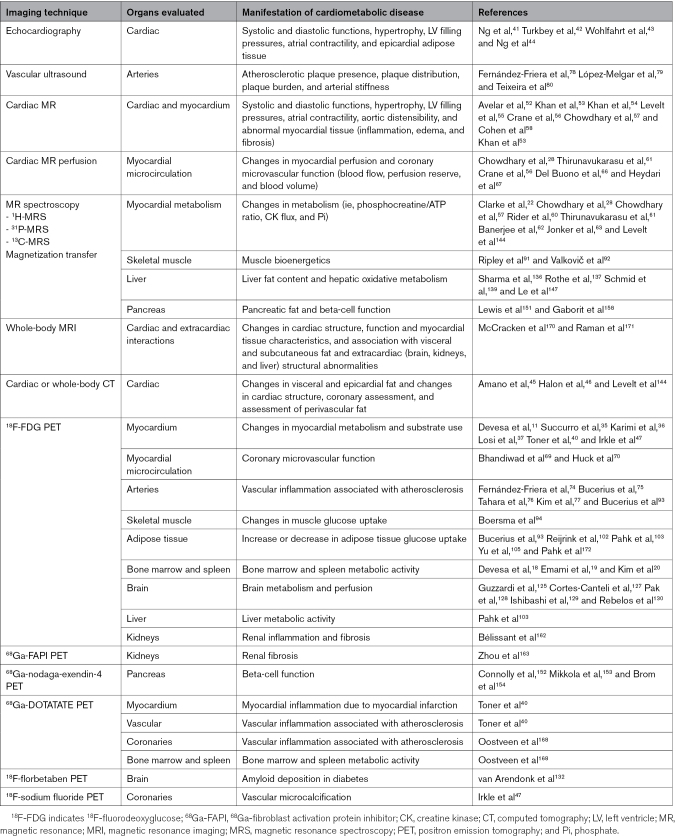
Contributions of Each Imaging Technique to the Evaluation of Cardiometabolic Diseases

## Connections Between Organs

### Imaging Evaluation of Connection Between Organs

The connections between organs in cardiometabolic diseases are essential for understanding the broader pathophysiology.^2^ These interactions often extend beyond individual organs, provoking cascading abnormalities that can contribute to the development and progression of diseases.^2^ Imaging techniques, alongside molecular biomarkers, can help reveal these connections and detect early cardiometabolic disease risks^173^ (graphic abstract). Hybrid imaging techniques, such as PET/CT and PET/MRI, are useful in studying interorgan crosstalk. For example, in the PESA study (Progression of Early Subclinical Atherosclerosis), a combination of PET/MRI with other techniques such as CMR, cardiac CT, and vascular ultrasound provided detailed insights into the interactions between systemic atherosclerosis, insulin resistance,^6,174^ metabolic risk factors, and various organs,^18^ including the brain,^127^ heart,^7,11^ and adipose tissue.^175^ Studies using these techniques have also been instrumental in understanding the brain-heart axis,^176^ adipose tissue–atherosclerosis link,^172^ and more, as they provide a holistic view of multiple organ systems affected by cardiometabolic diseases. In addition to hybrid modalities, the combination of different imaging techniques allows the study of the interactions between organs. For example, the MESA (Multi-Ethnic Study of Atherosclerosis) has used multimodality imaging, such as brain and CMR, coronary and chest CT, carotid ultrasound, and echocardiograms, to explore how cardiometabolic risk factors impact various organs and their interactions.^177–181^ Furthermore, the study of metabolic pathways between organs has benefited from MRS, which shows promising results for understanding interorgan metabolic interactions.^182^

### Examples of Connections Between Organs

#### Heart-Liver Axis

The heart-liver axis represents a complex, bidirectional relationship driven by shared metabolic dysfunction, systemic inflammation, and neurohormonal dysregulation. Both cardiometabolic HF with preserved ejection fraction (HFpEF) and MASLD are linked by obesity, insulin resistance, hypertension, and dyslipidemia, which contribute to cardiac and hepatic pathology. Emerging evidence suggests that MASLD may independently drive metabolic heart disease through direct interorgan crosstalk, beyond traditional cardiometabolic risk factors. One key mechanism is chronic low-grade inflammation, where MASLD promotes a proinflammatory milieu through elevated cytokines such as interleukin-6, tumor necrosis factor-α, and C-reactive protein, in turn contributing to systemic endothelial dysfunction, vascular stiffness, and microvascular disease, all of which are central to HFpEF progression. In addition, MASLD-induced oxidative stress worsens cardiac mitochondrial dysfunction, increasing the risk of diastolic dysfunction.^135^ Hepatic metabolic dysregulation also plays a pivotal role in cardiac substrate utilization. MASLD leads to increased very-low-density lipoprotein secretion, elevated free fatty acids, and hepatic insulin resistance, driving myocardial steatosis and lipotoxicity. This shift forces the heart to rely excessively on fatty acid oxidation at the expense of glucose metabolism, contributing to metabolic inflexibility and energy depletion, hallmarks of HFpEF. In addition, impaired ketone metabolism in MASLD may deprive the heart of an alternative fuel source, further exacerbating energetic deficits. Beyond metabolic substrates, liver-derived secretory proteins (eg, fibroblast growth factor-21, fetuin-A, and adropin) have also been implicated in cardiac inflammation, fibrosis, and insulin sensitivity. Liver-derived extracellular vesicles enriched in microRNAs (eg, miR-122) may further mediate myocardial remodeling and mitochondrial dysfunction.^183,184^

Among patients with diabetes and HFpEF, studies have demonstrated associations between diastolic dysfunction and elevated liver fat fraction or fibrosis.^185^ Patients with HFpEF and MAFLD show more advanced liver fibrosis, including cirrhosis, compared with those with MAFLD alone.^186^ Conversely, individuals with liver disease often have a higher burden of cardiac abnormalities, with the severity of liver disease correlating with increased myocardial edema and fibrosis.^187^ A UK Biobank study of 33 616 participants found that liver inflammation and fibrosis were associated with a doubling of cardiovascular risk, even in individuals without overt metabolic risk factors.^145^ Hepatic fat, quantified via CT and MRI,^144^ may enable early identification of MAFLD and metabolic dysfunction–associated steatohepatitis, conditions associated with heightened risk of cardiovascular events.^188,189^ However, while liver fat is strongly tied to cardiometabolic conditions, its direct association with cardiac outcomes remains unclear, with inflammation likely playing a more significant role.^145^

#### Muscle, Fat, Microcirculation, and HF

The interplay between muscle, fat, and microcirculation is integral to cardiovascular disease, particularly in HFpEF.^190^ Excess visceral and epicardial fat contributes to systemic inflammation and vascular stiffness, increasing cardiac workload and impairing myocardial relaxation.^191^ Adipose tissue–driven inflammation disrupts microvascular function, exacerbating coronary microvascular dysfunction, which limits oxygen delivery, creating an oxygen supply-demand imbalance that impacts cardiac efficiency. Skeletal muscle dysfunction further compounds this issue. In HFpEF, fat infiltration into muscle reduces oxidative capacity and perfusion, leading to early fatigue and diminished exercise tolerance.^192^ This muscle weakness, exacerbated by fat infiltration, impairs the muscle’s ability to adequately extract oxygen, amplifying cardiovascular stress as the heart compensates for the decreased muscle perfusion. Consequently, this cycle of inflammation, impaired microvascular function, and reduced muscle capacity perpetuates HFpEF symptoms, reducing the quality of life.

Beyond HFpEF, the interplay between muscle and adipose tissue has systemic implications. Altered glucose uptake in skeletal muscle, visceral fat, and the brain is linked to whole-body insulin resistance, highlighting the interconnected metabolic pathways driving cardiometabolic diseases.^94^

#### Brain-Heart Axis

The heart-brain relationship in MetS is complex and bidirectional, with advanced imaging revealing numerous links.

The autonomic nervous system, critical for heart rate and blood pressure regulation, often exhibits dysautonomia in cardiometabolic diseases, manifesting as sympathetic overactivity or impaired parasympathetic function.^193^ Functional MRI studies highlight altered connectivity in brain regions regulating autonomic control in MetS and diabetes.^194^ Structural MRI has revealed reduced white matter connectivity in these populations, correlating with increased cardiovascular risk. In addition, cardiac conditions such as atrial fibrillation and HF are linked to neurological complications, including stroke and cerebral hypoperfusion,^195^ which may further impair cognitive function. Studies from the UK Biobank have also demonstrated correlations between adverse cardiac phenotypes, such as increased aortic stiffness, and brain abnormalities, such as white matter hyperintensities, emphasizing the interconnectedness of cardiac and neural health.^170^

The use of DCE-MRI has allowed the identification of increased blood-brain barrier permeability following myocardial infarction in both experimental and clinical studies.^196^ This phenomenon has also been observed after ischemic stroke, and the underlying mechanism appears to involve the release of inflammatory factors that compromise blood-brain barrier integrity. This disruption plays a significant role in the development of neurological complications following myocardial infarction.

#### Kidney-Heart Axis

The heart-kidney axis is a well-established area of research, highlighting the interdependence between these organs in health and disease.^197^ Hypertension, diabetes, and microvascular disease often simultaneously affect both organs, with endothelial dysfunction playing a central role.^198^ Imaging is an important tool for assessing the function of both organs. In cases of chronic kidney disease, CMR parameters, including myocardial strain, T1 and T2 mappings, show significant alterations that worsen as chronic kidney disease progresses.^199^ Cardiac CT can also provide valuable information on cardiac and vascular calcification and relevant parameters in uremic cardiomyopathy.^200^ Furthermore, PET imaging studies have demonstrated that even moderate chronic kidney disease may accelerate a decline in coronary flow reserve.^201^

#### BM, Vascular Disease, and Ischemic Events

The BM plays a significant role in the interaction between cardiometabolic risk factors and cardiovascular diseases. Increased BM activity in individuals with MetS is linked to elevated inflammatory markers, contributing to early atherosclerosis, as evidenced by increased arterial ^18^F-FDG uptake and the presence of atherosclerotic plaques.^18^ BM activity also intensifies following acute cardiovascular events, such as myocardial infarction, with concurrent splenic activation.^19,20^ PET/MRI studies have further shown that increased BM activity correlates with decreased myocardial glucose uptake, reducing myocardial efficiency.^11^ These findings emphasize the BM’s critical role in both chronic atherosclerosis and acute ischemic events.

#### Spleen-Heart Axis

The spleen’s role in modulating the immune response after acute cardiovascular events is crucial.^202,203^ Several studies both in animals and humans have shown that there is an increase in the metabolic activity of the spleen occurring after myocardial infarction or stroke.^19,202^ Moreover, ischemic preconditioning experiments have demonstrated the spleen’s cardioprotective function. During acute events, the spleen releases cardioprotective factors that reduce infarct size, highlighting its importance in cardiovascular health and recovery mechanisms.^204^ These findings support the spleen’s potential as a therapeutic target in heart disease.

## Role of Imaging in the Management of Cardiometabolic Diseases

Imaging techniques play an important role in the management of cardiometabolic diseases. Cardiac CT facilitates the monitoring of statin therapy by characterizing atherosclerotic plaques, analyzing their composition, and identifying high-risk features.^50,51,107,113^ Furthermore, perivascular FAI assessed via CT decreases following statin therapy, enabling the tracking of reduced vascular inflammation in response to treatment.^205^

Therapies such as SGLT2 inhibitors and GLP-1 receptor agonists in patients with obesity and diabetes can also be monitored using advanced imaging modalities. CMR provides insights into the effects of these therapies on myocardial perfusion and cardiac function.^57,58^ In addition, MRS offers a means to evaluate cardiac metabolic response to these treatments^57,61^ and assess the functional response of pancreatic β cells.^159,206^

Imaging can also detect cardiometabolic changes induced by drugs. Several beneficial drugs can significantly alter cardiovascular metabolism; among the most notable are cancer treatments. Anthracyclines, commonly used as first-line therapy, are linked to cardiovascular complications in up to 30% of patients.^207^ Experimental studies using PET have shown that even at low cumulative doses, anthracyclines alter cardiac substrate utilization, inducing severe alteration in cardiac energetics.^38^ Anthracyclines induce severe mitochondrial damage, resulting in early intracardiomyocyte edema. The combination of T2 mapping and precontrast/postcontrast T1 mapping can noninvasively detect these early cardiac changes induced by anthracyclines.^208^

## Future Perspectives

Imaging modalities are rapidly advancing, along with our understanding of the consequences of MetS and its associated conditions on various organs. However, critical gaps remain, necessitating further research to fully elucidate the effects of cardiometabolic conditions across different systems.

A major limitation of current studies is their predominantly cross-sectional design, which does not establish cause and effect. A deeper understanding of how dysfunction in one organ contributes to susceptibility in others is critical, particularly in cardiometabolic diseases where organ interdependence can amplify disease progression. For example, how liver fibroinflammation may predispose the heart remodeling or neurovascular changes in the brain remains incompletely understood. Similarly, identifying individuals who are predisposed to multiorgan involvement could help develop earlier, more effective interventions.

In this context, multidisciplinary clinics and collaborations that manage diseases spanning the heart, brain, and liver in individuals with MetS are urgently required. Such clinics should include clear referral pathways to specialist care and foster teamwork among cardiologists, hepatologists, neurologists, and endocrinologists. By addressing multiorgan dysfunction holistically, these clinics could significantly improve patient outcomes in cardiometabolic diseases.

Another important avenue for research is the identification of preclinical imaging markers that detect organ dysfunction even before structural, irreversible changes occur or blood tests become abnormal. Early identification of these substrates could highlight opportunities for reversibility and treatment. Longitudinal imaging studies and clinical trials are needed to confirm that cardiometabolic treatments can not only improve metabolic states but also reverse imaging-detected abnormalities in a favorable manner. These studies would provide critical insights into the potential for regeneration and recovery of organ function.

In addition, most current imaging focuses on static assessments, which limits our ability to evaluate dynamic physiological changes. Developing more nuanced, reproducible, and widely available imaging modalities that can assess metabolism and cardiac physiology in real time is vital. Dynamic imaging during metabolic challenges, exercise, or following a vasoactive stimulus could reveal key insights into the mechanisms of disease and response to therapies. However, existing advanced imaging technologies (eg, hyperpolarized carbon-13, PET/MRI, PET/CT, and multinuclear MRS) remain cumbersome, expensive, and with limited global availability. There is also a shortage of expertise in these techniques, an incomplete understanding of potential confounders, and insufficient biological correlation with clinical outcomes. Addressing these gaps remains a priority for the future and will require technological innovation, training, standardization of protocols, and biological validation to make these tools more clinically accessible.

Finally, the concept of multiorgan interdependence highlights the need for a holistic approach. Despite the significant advances, many gaps still remain in understanding the interplay between various organ systems. Specifically, cross-organ talk involving the kidney, spleen, pancreas, and other organs remains underexplored underscoring the need for further research. For example, nuclear imaging holds promise for assessing renal and pancreatic changes in cardiometabolic conditions, yet its utility in routine practice requires further validation. Similarly, the impact of insulin resistance on the arterial system and atherosclerotic risk could provide valuable insights into cardiovascular event risks in MetS. To advance this field, hypothesis-driven research, longitudinal studies, and clinical trials focused on therapeutic impacts on multiorgan imaging biomarkers are crucial with the potential to predict, prevent, and treat complex cardiometabolic diseases more effectively.

## Conclusions

In conclusion, cardiometabolic diseases represent a complex interplay of metabolic, inflammatory, and cardiovascular abnormalities that affect multiple organs, including the heart, brain, liver, kidneys, skeletal muscle, adipose tissue, and hematopoietic system. The disruption of interorgan crosstalk amplifies disease progression, contributing to significant morbidity and mortality. Advances in imaging modalities, such as MRI, CT, PET, and hybrid techniques, such as PET/CT and PET/MRI, have generated vital insights into organ-specific changes but also enabled the evaluation of systemic interactions, such as the brain-heart, heart-liver, and fat-muscle-heart axes, highlighting the pivotal role of interorgan communication. By assessing the structural, functional, and metabolic alterations associated with cardiometabolic diseases, imaging can facilitate early diagnosis, risk stratification, and monitoring of disease progression and treatment effects. As the field evolves, integrating these advanced imaging approaches with clinical practice will be essential for developing targeted therapies that address both organ-specific and systemic mechanisms underlying cardiometabolic diseases.

## ARTICLE INFORMATION

### Acknowledgments

The graphic abstract was created with BioRender (https://BioRender.com).

### Sources of Funding

The Centro Nacional de Investigaciones Cardiovasculares (CNIC) is supported by the Instituto de Salud Carlos III, the Ministerio de Ciencia, Innovación y Universidades (MICIU), and the Pro CNIC Foundation and is a Severo Ochoa Center of Excellence (grant CEX2020-001041-S funded by MICIU/AEI/10.13039/501100011033). A. Devesa is scientifically supported by the Thrasher Research Fund and acknowledges the support of Sociedad Española de Cardiología (grant SEC/PRS-MOV-INT20/002). B. Ibanez is supported by the European Commission (grants ERC-CoG 819775 and H2020-HEALTH 945118), the Spanish Ministry of Science and Innovation (grant PID2022-140176OB-I00), the Red Madrileña de Nanomedicina en Imagen Molecular-Comunidad de Madrid (grant P2022/BMD-7403 RENIM-CM), and a grant from the Novo Nordisk Foundation, REACT. B. Raman is funded by a Wellcome Career Development Award Fellowship (grant 302210/Z/23/Z). E. Nagel receives funding from the German Centre for Cardiovascular Research (DZHK) (grant 81Z0200109). L. Valkovic is funded by a Sir Henry Dale Fellowship supported jointly by the Wellcome Trust and The Royal Society (grant 221805/Z/20/Z) and acknowledges the support of Slovak Grant Agencies VEGA (Vedecka grantova agentura MSVVaM SR a SAV; grant 2/0004/23) and APVV (Agentura na Podporu Vyskumu a Vyvoja; grant 21-0299). B. Raman is also supported by the NIHR Oxford BRC (Multiorgan Imaging Theme).

### Disclosures

V. Delgado received speaker fees from Abbott Structural, Edwards Lifesciences, GE Healthcare, JenaValve, Medtronic, Novartis, Philips, Products & Features, and Siemens and consulting fees from MSD, Novo Nordisk, and Edwards Lifesciences. E. Nagel received research funding and consultancy fees from Bayer AG and is the founder of Goethe CVI GmbH. The other authors report no conflicts.
